# Spirit of the British and American Periodicals

**Published:** 1838-10-01

**Authors:** 


### Spirit of the British and American Periodicals.

DR. GRAY'S UTERO-ABDOMINAL SUPPORTER—AN IMPROVEMENT ON THAT OF DR. HULL.

WE have received the Transactions of the Medical Society of New York, Vol. 4, Part 1., in which is a long paper by Dr. Gray, on Prolapsus Uteri. The observations are so close and connected that we are unable to give any regular analysis of the paper. This is of less consequence, as any of our medical brethren may examine the apparatus at Dr. Gray's apartments, 279, Regent Street. We insert, however, the following stringent remarks on the old and new treatment of prolapsus uteri.

" The indications of cure growing out of this view of the nature of prolapsus, are :

I. To diminish the preternatural capacity of the hypogastric, and ano-perineal regions ; and,

II. To restore tone to the muscular portions of those parts.

The first of these indications is answered by the ingenious apparatus of Dr. Hull, entitled by him, *Utero Abdominal Supporter*, which presses against the two weakened and distended points of the abdomen, with any required force, and at the same time preserves by its elasticity, the faculty of advancing and receding with the respiratory action of these parts. The second indication is also fulfilled to some extent by this instrument, by means of the well known sanatory influences of equal and uniform compression upon parts distended and weakened by forces from within. At any rate, it affords the weakened muscular and aponeurotic apparatus it covers, rest and opportunity to recover their lost tone ; an object of so much importance in the estimation of Prof. Burns, Dr. Davis, and many other eminent writers on the subject, as to induce them to insist upon nearly absolute rest in a recumbent posture for a very long period of time.

I have had frequent opportunities during the past four or five years, of seeing the results of the late Dr. Hull's instrument, and they have been such as fully to justify the views I have set forth, and to convince me that the old and ever inefficient expedients of the recumbent posture, styptic injections, pessaries, &c., and the erroneous views upon which they are founded, will be exploded in a very short time.

The objections to the old practice become more apparent and palpable, when considered in connexion with the views I have advanced.

I. *The Horizontal Posture.*—This is earnestly insisted upon by Prof. Burns, Sir Charles Clarke, M. Gardien, and Dr. Davis of London, the most eminent recent writers on obstetric medicine. It is founded on the hypothesis of the ligaments, and cannot, it appears to me, prove serviceable in any great number of cases. It is possible that, in some instances, presenting great vigor of muscle, and fullness of the vascular system, the loss of appetite and general relaxation necessarily resulting from a rigid adherence to this irksome position, for two or three months, the shortest time named by Dr. Davis, might, by greatly reducing the upper forces of the abdomen, palliate the prolapsus to a considerable extent. But though the recumbent posture immediately relieves the patient from the dragging and bearing down which are especially distressing in the early appearance of the displacement, it is not to be forgotten, that a large majority of those subject to this malady, are, by no means, in a condition to sustain, without serious consequences, the prostration and general debility caused by persisting in it for any length of time. Indeed, the authors cited, one and all, condemn this treatment, in principle, by strongly insisting upon the use of means for invigorating the system, a loss of tone in which they consider the remote cause of the disease. Dr. Davis affirms that the horizontal posture, ' rigidly observed for two or three months, might possibly suffice to insure a restoration of the suspensory ligaments [which are they ?] of the uterus to their former tone and strength.' He offers no experience whatever in support of the prescription, nor does either of the other writers on the subject. It appears to be founded solely upon the prevalent but irrational supposition, that the peritoneal folds and round ligaments suspend the uterus, in the healthy state, and that relaxation in them is the proximate cause of prolapsus ; and this pursuit of a hidden cause, which transcends the possible limits of testimony, in this, as in very many other cases in medicine, seems to take off the attention from the plainest possible mandates of experience.

Professor Thompson asserts that this practice 'tends not only to impair the general health, but also to aggravate the disease, by *increasing the relaxation of the natural supports of the womb ; daily experience has established the validity of this opinion.*' Mad. Boivin does not recommend its adoption.

II. *Styptic Injections into the Vagina.*—This practice, so frequently followed by pernicious consequences in the treatment of leucorrheal discharges, is also recommended by the eminent authors above quoted.

It is difficult to conceive how the injection of astringents into the vagina, can restore tone to the round ligaments, or to the peritoneal folds, or corroborate their convalescence ; yet such are the faculties ascribed to them. But it is easy to imagine that they may irritate the mucous membrane of the vagina, and suddenly dry up habitual discharges from it, to the very great detriment of the general health ; and also excite serious indurations, and even ulcerations of the os uteri. Much indubitable testimony exists in the records of medicine to support the latter supposition, but so far as I am aware, none whatever in favor of the former. Is it not probable that both these expedients, the horizontal posture and the use of styptics, have been resorted to, successively, and after other means, because practitioners have been so constantly baffled in the treatment of the malady in question ?,

III. *Pessaries.*—After permitting the descent of the intestines upon the viscera to take place, without pathological recognition, it is not to be wondered at that the appearance of the uterus at the vulva should be regarded by the earliest surgeons as the result of relaxation of its upper connecting points; nor that its farther descent should be opposed by the introduction of a prop, or block, into the vagina, which, upon having its long diameter arranged across that passage, should be incapable of expulsion through its outlet, thus presenting an effectual barrier against the exit of the womb from that part. But it is matter of surprize, as before stated, that the improvements in anatomical knowledge which the last century has developed should not have produced a better mode of curing prolapsus than was devised in the first and rudest stage of surgical invention.

The objections to the application of pessaries are :

1. They are not surgically indicated; they consequently never act as remedies, but merely palliatives in all cases, and like all other palliatives, they tend to perpetuate the necessity of their application during the life of the patient.

2. They distend the flooring of the pelvis and increase the capacity of the ano-perineal region, thus directly aggravating one essential and important element of the disease.

3. They provoke mucous discharges from the vagina, where they do not exist, and change the character and quantity of existing ones, much for the worse.

4. They occasionally produce serious inflammations. Velpeau relates a fatal peritonitis, produced by a pessary, introduced by himself.—*Surg. Anat.* ii. p. 283.) They cannot be worn by many patients on account of the local and constitutional irritation they produce.

5. They are likely to hasten the development of latent scirrhous affections of the os uteri, which are sometimes complicated with prolapsus, and perhaps more frequently than has been suspected. (S. Cooper quotes cases from Ruysch & Langenbeck, in his *Surgical Dictionary,* art. Prolap. Uteri.)

This is a very strong objection to pessaries.

6. They interrupt sexual intercourse.

7. Numerous instances are related in surgical works, of pessaries becoming so encrusted and firmly imbedded in the vagina, as to require serious operations to extract them.

8. Their proper application, the choice of their form and dimensions, so as to adapt them well to each case, is a point of much difficulty with the most expert surgeon. The majority of practitioners must find extreme difficulty in these respects.

The unsatisfactory results of ordinary methods, recently induced a practitioner of high character to undertake a radical cure, by obliterating the cavity of the vagina! Mr. Liston brought the sides of the vagina together by several ligatures. It is scarcely necessary to say, that this cruel experiment failed entirely. Professor Thompson sanctioned the attempt.

Dr. Marshall Hall has lately removed a strip of the mucous membrane, an inch and a half wide, from the whole length of the vagina, for the purpose of contracting the diameter of that canal so much as to prevent the descent of the womb through it. I mention these cases, not by any means as precedents worthy of imitation, but as showing conclusively the inefficiency of the prevailing practice in this disease, and the vagueness of the views upon which such practice is founded.

The method of Dr. Hull is liable to none of the objections enumerated, nor so far as I am able to judge is it objectionable in any regard. A large number of my professional colleagues in New-York have applied the new apparatus, and all, I believe, with gratifying success.

There is an important consideration in favour of the external mechanical support worthy of notice; this is its salutary influence in the flexions and lateral displacements in the fundus uteri not accompanied by prolapsus. It is probable, as before stated, that retroversion, anteversion, and the lateral dislocation of Meckel, are caused by a relaxation of the hypogastrium, permitting the small intestines to sink below the brim of the pelvis. The circular portion of Dr. Hull's Supporter would in this case be indicated, and doubtless prove successful. Farther experience will determine how far abortions from this condition may thus be averted. It is probable also that many distressing gastric affections, arising from dislodgement of the intestines, and ascribed to other causes, and particularly to morbid sympathy with the uterus, may be effectually removed by this apparatus. The same remark may be made in reference to affections of the urinary bladder. I have seen two cases of partial incontinence of urine, not connected with prolapsus, cured by this instrument. It was applied upon the supposition that the intestines compressed the bladder and diminished its capacity. The distended condition of the hypogastrium and hollowing of the upper part of the belly before spoken of, existed in both instances, and gave rise to the conjecture as to a mechanical cause."

We understand that Drs. Conquest, Blundell, and others have given their sanction to the utero-abdominal bandage; but can give no opinion of our own.

---

### CASE OF DIABETES MELLITUS IN A BOY OF NINE YEARS OF AGE, WITH THE APPEARANCES ON DISSECTION. By JAMES JOHNSON, M.D.

A fine boy was severely affected, a few years ago, with what is termed bilious remittent fever—or more properly a gastro-intestinal fever, which nearly destroyed his life. After this I lost sight of him for two or three years. I was called to him on the 15th June 1838, by Mr. Clifton, who was in attendance upon him for diabetes. This disease had been detected only a few weeks previously, and the boy, from being a fine active youth, was getting thin and emaciated. The thirst was great, and he passed eight or ten pints of water, very sweet, and of high specific gravity. No tangible or appreciable disease could be detected in any part of the body. The tongue was white, and the pulse too quick. He was placed on animal food diet,—Dover's powder with hyd. cum creta—and the warm bath. Under this plan he seemed to stem the effects of the disease; but little or no alteration took place in the diabetes. He was cupped over the region of the kidneys. The general health certainly did not deteriorate. Dr. Bright saw him in consultation with myself and Mr. Clifton, and recommended some slight additions to the means abovementioned. The mother of this unfortunate youth was persuaded by some friend, at this time, to try a remedy which was said to have proved effectual in a desperate case of diabetes—and this was, the exhibition of the patient's own urine for drink. Unknown to the medical attendants, the beverage was given to the boy, and, strange enough, he relished it exceedingly, being informed that it was a diet-drink sent into town from the country! For at least ten days he drank the whole of his own urine; but still, the quantity excreted was greater than the quantity of urine drunk, because there were other fluids also taken besides the urine. The mother, who was rather more credulous than judicious, fancied the boy improved in health during this time; but such was not the case. She confessed the truth, and the urinous drink was discontinued. It is worthy of remark, however, that the boy appears to have discovered the nature of the diet-drink; for he was, more than once afterwards, found by the nurse drinking his own urine.

About this time the boy's mother was strongly urged to consult an eminent homœopathist (whose name I shall not mention) and as he promised to cure the patient, a promise which the allopathists would not make, he was employed. As I did not interfere in the new treatment, I only occasionally called to see the proceedure, and always met the disciple of Hahneman at my visits. He was giving the boy some millionth doses of belladonna when I first met him. I asked him if he was proceeding on the established canon of homœopathy—" similia similibus curantur "—and he replied in the affirmative. I asked him what medicine would produce diabetes mellitus in a healthy subject? He hesitated for some time, and then affirmed most positively that belladonna would cause diabetes!! After this I had no more arguments on the subject. It is hardly necessary to say that the boy gradually, but steadily wasted, and died on the 28th of July.

The body was examined,* and the following notes were made. Body extremely emaciated. The thoracic viscera sound. The intestines were bound together at various points, making it difficult to trace their natural course. Their mucous membrane was highly vascular and discoloured for a considerable extent—in some places being of a greyish—in others of a brown hue. The mesenteric glands were enlarged and vascular. The stomach, liver, and spleen were healthy. The gall-bladder was empty. The kidneys were enlarged, and very turgid with blood, more especially the left kidney, around which the cellular membrane was rather loaded with a sort of gelatinous substance. The coats of the bladder were somewhat thickened.

It is hardly necessary to remark that homœopathy was not likely to do any good in this case; but the confident promises of success were not the less on that account. This instance only adds one to many others that have come under my notice, to convince me that homœopathy is a humbug.

We have no sufficient data on which to ground the assumption that diabetes mellitus is a disease dependent on disordered function or structure of any particular organ. I have found the lungs more frequently diseased than any other viscus in this grave malady. In the present case, however, the kidneys were the parts most affected, since the mucous membrane of the bowels became inflamed in the course of the diabetes, and presented the usual phenomena of that affection. It is certainly not a little curious that this patient should have drank his own urine for eight or ten days with avidity—and, for the first few days, with some slight apparent advantage.

* The *post mortem* was made in the country, and I have mislaid the name of the medical gentleman.

### CLIMATE OF TORQUAY.

Dr. De Barry, of Torquay, has been at some pains to collect and construct thermometrical tables, shewing the comparative climates of that place, and several other localities, during the months of January and February of the present year. The following summary Table will be sufficient for our purpose.

MEAN TEMPERATURE.

| | JANUARY. | | | |
|---|---|---|---|---|
| | Maximum | Minimum. | 9, a. m. | 9, p. m. |
| Torquay .. .. .. .. .. .. | 40.2 | 32 | 35.8 | 34.6 |
| Exeter .. .. .. .. .. .. | 36.7 | 27.7 | 31 | 30.7 |
| Royal Society, Somerset House | 34 | 26.6 | 29.5 | .. |
| Bristol .. .. .. .. .. .. | 34.5 | 25.4 | .. | .. |
| Edmonton .. .. .. .. .. | 32.3 | 18.5 | .. | .. |
| Sheffield .. .. .. .. .. .. | 51.1 | 24.5 | 26.6 | .. |
| Cheltenham .. .. .. .. .. | .. | .. | .. | .. |
| Farnham.. .. .. .. .. .. | .. | 24.6 | .. | .. |
| Torquay from Jan. 8th to 31st inclusive | 38 | 30.4 | .. | .. |
| Cheltenham ditto ditto .. | 31 | 23.8 | .. | .. |
| Torquay from Jan. 8 to Feb 4 inclusive | .. | .. | 33.6 | 33.1 |
| Hyde Park ditto ditto .. | .. | .. | 24.5 | 25.3 |
| | FEBRUARY. | | | |
| Torquay .. .. .. .. .. .. | 42.4 | 35.2 | 38.6 | 38.8 |
| Exeter .. .. .. .. .. .. | 39.7 | 31.6 | 34.8 | 35.6 |
| | .. | .. | 33.1 | .. |
| Bristol .. .. .. .. .. .. | 39.5 | 29.6 | .. | .. |
| Edmonton .. .. .. .. .. | 37.5 | 24.6 | .. | .. |
| Sheffield .. .. .. .. .. .. | 32.2 | 24 | 27.7 | .. |
| Cheltenham .. .. .. .. .. | 36.2 | 29.6 | .. | .. |

### NITRATE OF SILVER IN CHRONIC DISEASES.

Dr. O'Bryen has published a valuable paper in our Dublin contemporary on this subject. Professor Lallemand, of Montpellier, has been very successful in chronic cystitis, by touching the internal surface of the bladder with solid nitrate of silver. The following is the mode of application :—

" Monsieur Lallemand uses an instrument of the following description, indeed he himself chose this one for me. It consists of a large catheter (of pure silver, as, if there is any alloy, the caustic acts upon it) open at both ends, having two sorts of stilet, according to the part you wish to cauterize; at the extremity of each stilet is a small excavation, containing the caustic, which is first pulverized, and then placed in the excavation over a spirit lamp, which fuses and moulds it to the cavity.

When the instrument is prepared, introduce into the bladder an ordinary catheter, in order to empty it completely. This *precaution is strictly necessary,* for the urine would dissolve the caustic, and prevent its directly affecting the mucous membrane: when this has been withdrawn, the instrument bearing the caustic is to be introduced, (closed,) and the moment it has entered the bladder, you are to push the stilet, and rapidly turn the *porte caustique* from side to side two or three times, and then pull the stilet into the instrument, and withdraw it; our object should be to touch the surface in as many points as possible. While the instrument is within, the bladder contracts and grasps it, the kidneys secrete a small quantity of urine, as the lachrymal gland secretes tears when the conjunctiva is cauterized; but this small quantity of liquid, far from being hurtful, is on the contrary favourable, as it acts as a vehicle to the portion it does not decompose, and conveys it equally over the surface of the membrane. The patients feel, at that moment, a sharp pain at the neck of the bladder and in the rectum, described by them as not unlike a pinch, but much more supportable than the continued dull pain of chronic catarrh; there is now an irresistible desire to pass water, and as the bladder is nearly empty, very little passes, and this causes a burning along the urethra, and is accompanied by some drops of blood. This desire is renewed every moment, causing violent but useless efforts. These gradually decrease, and on the second and third day there is no longer any pain on making water, and a few small grey eschars, like burned paper, come away with the urine. This occurs in a large number of patients, but in some more susceptible, the process does not proceed so simply, particularly if you have used the *porte caustique* too long. In this case retention of urine follows, which lasts from three to thirty-six hours; even here we must not be in too great a hurry to use the catheter, as a warm bath, a few narcotic lavements, emollient drinks, some tartrate of soda, with infus. sennæ, and sometimes a few leeches, will cause the spasms to yield; if not, some belladonna to the meatus may be tried, always taking care to use antiphlogistics with moderation in the beginning, as inflammation is necessary to the cure. In a majority of cases one cauterization is sufficient to procure a cure; when it happens otherwise, a second and even a third application may be necessary, but Monsieur L. states that he never saw a case requiring a fourth.*

* Dublin Journal, Sept. 1838.

### On the State of the Pupil in Typhus, and the Use of Belladonna in cases in which the Pupil is contracted. By *Robert J. Graves*, M. D.*

Dr. Graves remarks that, in sleep, the pupil is contracted—that, in some diseased conditions of the brain the pupil is contracted, while, in the other, it is dilated, without our knowing the precise circumstances on which the one state or the other depends—that:—

" In fever with cerebral disease, one of the most alarming symptoms is marked contraction of the pupil, and were I called to a case in which every other symptom was favourable, but great contraction of the pupil present, I would say that it was a case of extreme danger. A tendency to even moderate contraction of the pupil is a very dangerous symptom in typhus, but a pupil extremely and permanently contracted, or as it has been called a *pin-hole pupil*, is, or used to be, a fatal sign. Although this symptom is so obvious, so easily ascertained, and unhappily so common, for I have within the last few years witnessed it in a great number of fatal cases, yet, strange to say, it has not attracted the attention it deserves, among writers on typhus."

That opium has the effect of occasioning contracted pupil, while stramonium and belladonna occasion dilated pupil; that opium scarcely ever fails to be injurious in cases of fever, with contracted pupil, even though the patient be restless, and greatly in want of sleep:—

" I have often seen it tried, and I think scarcely ever without more or less injury to the patient. When opium is administered to a patient in the advanced stage of fever, with symptoms of cerebral derangement, and tendency to contraction of the pupil, you will find that the pupil which has been moderately contracted to-day, will be greatly contracted to-morrow, and that the patient will soon sink into an irrecoverable state of coma. This contracted state of the pupil may exist in its ex-

* Dublin Journal, July 18, 1838.

treme and most marked form in typhus fever, without being necessarily accompanied by head-ache and delirium; the patients are restless, sleepless, and in a state of remarkable nervous excitement; but they answer questions not unfrequently in a tolerably clear and rational manner, and many of them distinctly affirm that they have no pain in the head. These circumstances may deceive the unwary, but the experienced practitioner, who has witnessed many such cases, will feel that a fatal termination is threatened. Under these circumstances, opium in every shape is injurious, and even tartar emetic fails in controlling or diminishing the pernicious effects of opium. This is somewhat curious, as the combination of tartar emetic and opium seldom fails in relieving cases similar in all respects, except the symptom of contraction of the pupil."

"That, it may appear somewhat extraordinary to think of prescribing for a single symptom, and giving belladonna in a case of fever with contraction of the pupil, merely because it produces dilatation of the pupil; but, as was before observed, it is not unreasonable to suppose that the state of the brain which accompanies dilatation of the pupil is different from that which accompanies contraction, and if belladonna has an effect in producing that cerebral state which is attended with dilatation, it is not going too far to infer, that its administration may do much towards counteracting the opposite condition; neither is it unphysiological to conclude, that if a remedy be capable of counteracting or preventing *one very remarkable effect* of a certain morbid state of the brain, it may also counteract *other symptoms* connected with the same condition."

The result of the reflections and of the experiments of Dr. Graves is, that belladonna is of service in cases of cerebral excitement in fever, accompanied by contraction of the pupil. He thinks it may be usefully combined with all the known preparations of opium.

"This combination evidently saved the life of a gentleman in Parliament-street, whom I attended along with Mr. Sibthorp and Dr. Dwyer, and whose case was apparently hopeless. In the Meath Hospital, draughts consisting of black drop, belladonna, and tartar emetic, have been eminently serviceable; the extract of belladonna which I have used, is, properly speaking, an inspissated juice, and is so called in the last edition of the Dublin Pharmacopœia."

Dr. Graves relates the details of two cases in which belladonna appeared to be of service, and concludes by observing, that enough has been done to stimulate inquiry, and it will be granted by all, that there is no subject more deserving of the earnest attention of the physician than the treatment of cerebral affections in typhus fever. Where a tendency to contraction of the pupils accompanies this disease, the usual plan of blistering, leeching, cold lotions, &c., will prove insufficient. It may be of some use in the commencement, or in the middle stage of fever, but not towards the end, when the vital powers are excessively reduced, and the patient hangs on the verge of dissolution. The paper of Dr. Graves deserves, as all that proceeds from his pen does, the attentive consideration of practical men.

---

### BENEFICIAL EFFECTS OF BELLADONNA IN A CASE OF PUERPERAL MANIA. By DAVID H. SCOTT, M.D.*

We notice the following case because it bears indirectly upon the preceding paper.

A lady, the mother of four children, was confined of her last in April of the past year. Since her childhood she had been constitutionally nervous, and partaking of an hereditary excitability. About two months previously to the full period of gestation, she began to express a conviction that she should not recover, and, in spite of all persuasion, this increased. But her labour was favourable, and she went on well until the evening of the seventh day,

* Dublin Journal, July, 1838.

when her husband found her sitting up in bed, reciting with astonishing rapidity and accuracy several parts of scripture, and hymns, many of which she had learned in her childhood, and which had been apparently forgotton by her, as she was never, on any occasion before, known to repeat them, neither could she when requested to do so. She was quite unmanageable, scarcely recognized any person at her bed-side, answered incoherently the questions proposed, dwelt constantly on the idea of death, for which she fancied she had been so actively preparing, and though her medical attendant was instantly on the spot, it was with much difficulty he was able to apply his measures. Active purging had a beneficial effect, it relieved the urgency of the symptoms. At this time she was attacked with mammary abscess, and had been prohibited nursing the child. Her nights were now sleepless; opium was largely administered to give repose. She became abstracted, and shunned all society; a melancholy, with occasional paroxysms of high excitement, seized her mind; to those whom she dearest loved she showed the strongest dislike, and abandoned herself to despair and to utter ruin, an object unworthy of the compassion of her Creator. Leeches, occasional purgatives, change of scene and air, regulation of the secretions, which were greatly vitiated, the shower-bath, were employed with partial and indifferent benefit. But at the end of October there was an evident change, the intervals of active excitement became longer, and her manner more rational; she began to feel a pleasure in occasional conversation, and the cautious introduction of visitors seemed to draw her attention to other objects than the delusions which haunted her. Dr. Scott now inserted a seton in the nape of the neck. It only rendered her unmanageable, and was withdrawn. Thinking that direct action on the nervous system might be serviceable, Dr. Scott combined with perseverance in the use purgatives, a pill composed of half a grain of extract of belladonna to be taken every night.

The effect of this treatment was highly satisfactory, her nights were visited with refreshing sleep, her skin, which had been parched from the onset of the illness up to this period, became moist, symptom after symptom improved, and, at the end of six weeks, her eye lost its vacuity, her countenance wore a contented and intelligent expression, her mind was calm, collected, and happy, and her feelings so altered, when contrasted, as she expressed it, with her past wretched and dismal state. The belladonna was employed up to the period of her permanent improvement, which was toward the end of December, when, to use the language of her husband, " she was as well as she had ever been during her life."

---

### Facility with which the Natives of India recover from Serious Accidents.

In the India Journal, for April 1, 1838, we find a communication from Mr. Spilsbury surgeon of Jubbulpore, from which we extract the following passage.

" Some time ago I had occasion to bring to the notice of the Medical and Physical Society two remarkable cases of wounds, showing with what facility serious accidents are gotten over by the Natives. The first was, that of a grass-cutter gored by a Spotted Deer with protrusion of the intestines; the second that of a woman in the very last stage of pregnancy pitched on the horn of a bullock, which perforated the cavity of the uterus; to these serious ones of the abdomen and pelvis I have to add another.

A young woman about 18 was brought to me with a very extensive wound of the occiput and posterior portion of the parietal bones inflicted with a tulwar, a piece of the skull and margin was completely detached, laying bare the the brain to that extent; she had likewise a severe wound of the left hand cutting through the metacarpal bone of the fourth finger, also a very slight cut on the top of the same shoulder.

When brought to me she had lost a very considerable quantity of blood, her clothes being quite saturated, and was in a very exhausted state from the time of dressing her wounds, up to their healing, which they all did speedily, with exception of the apparently very trifling one on the shoulder, not the smallest symptoms of affection of the head took place, she never complained of headache nor was there the slightest tendency to heat of skin, except in the injured hand for a day or two. P. nat. app. good, sleep unimpaired, adding another instance of the little constitutional irritation that takes place in natives after very dangerous wounds."

---

OBSERVATIONS ON THE FLUID IN THE VESICULÆ SEMINALES OF MAN. By JOHN DAVY, M.D., F.R.S., Assistant Inspector of Army Hospitals.*

In this country opinions are unsettled with respect to the uses of the vesiculæ seminales. Though some persons think them reservoirs for the secretion of the testes, the majority adopt the opinion of Hunter, and conclude that they furnish a fluid subsidiary to the semen.

Dr. Davy has investigated the subject in a very able, and a very complete manner, and the result of his researches is contained in our esteemed northern contemporary. His paper is a long one, the observations contained in it are numerous, and the conclusious are sufficient for our space and purpose. The secretions of the testes, vesiculæ, and vasa deferentia, were examined in twenty cases.

Excepting in two instances, viz. the 18th and 20th, no animalcules could be seen in the fluid expressed from the divided substance of the gland. The fluid, when it could be obtained in sufficient quantity for accurate observation, was transparent, generally contained globules nearly equal in diameter to the blood corpuscles, and invariably contained dense particles, apparently spherical, very much smaller, from twelve to fifteen times smaller, and which, it may be conjectured, are the ova of the spermatic entozoa. In the two instances in which spermatic animalcules were found in the fluid of the tubuli, the quantity of the fluid was greater than in the others.

The inferences drawn from the whole of the observations are these :—

The first inference is that the *vesiculæ* are seminal reservoirs, according to the old opinion on the subject, and that which is still most commonly entertained by the continental physiologists. And next, that they are not merely reservoirs, but are also secreting organs, furnishing mucus, and perhaps some other fluid, for admixture with the semen.

The first inference is supported by the general resemblance, in several cases, of the fluid of the *vasa deferentia* and of the *vesiculæ*, and of the existence of the characteristic spermatic animalcules in the fluid of the *vesiculæ*, in every instance in which they were detected in the fluid of the *vasa deferentia*. Hunter does not mention having used the microscope in his inquiry. If he had, he could hardly have failed to have arrived at a different conclusion.

"The second inference is supported by there being a certain difference in almost every case between the fluid of the *vesiculæ* and that of the *vasa deferentia*, and especially by the circumstance, that the difference of quality is most perceptible in the fluid of the fundus,—where most out of the way of being readily mixed with the fluid of the testes. What the exact difference of qualities is between the fluid of the vesiculæ and of the *vasa deferentia*, and, it may be added, of the *vasa deferentia* and of the testes, in perfect health, remains to be ascertained. It can be determined only by careful examination and comparison in the instances of criminals who have been executed, or of persons who have been killed by accident, not labouring under chronic disease, and in the vigour of life. I am disposed to think that the difference will not be found very considerable, and that between the fluid of the vesiculæ and of the *vasa deferentia*, it will consist chiefly in the former being more dilute and perhaps more bland and mucous. Hunter was of opinion that the fluid of the vesiculæ is naturally of a brownish hue. As an invariable quality this appears questionable. The sooner after death the fluid is examined, the less brown it is. I have given amongst the preceding

---

* Edin. Med. & Surg. Journal, July 1, 1838.

cases, instances in which it was colourless, and those examples in which there was reason to consider it least altered in consequence of disease: and, Mr. Hunter's best observations are in accordance, viz. two instances, the only ones given in which examination was made very soon after death. He says, in a man killed by a cannon-ball, 'the fluid in the vesiculæ was of a lighter colour than is usually found in men who have been dead a considerable time; but it was by no means like the semen either in colour or smell.' And, he adds, 'in another man who died instantaneously in consequence of falling a considerable height, and whose body I inspected soon after the accident, I found the contents of the vesiculæ of a lightish whey-colour, having nothing of the smell of semen, and in so fluid a state as to run out on cutting into them.' The colour which the fluid of the vesiculæ commonly exhibits in late *post mortem* examinations is probably partly derived from the infiltration of some of the colouring matter of the blood after death, and, in part, perhaps occasionally, from a vitiated secretion of the follicles, or secreting surface of the vesiculæ, and of the adjoining *vasa deferentia*.

It would be a work of supererogation to enter into a detailed examination of the arguments brought forward by Hunter in support of the views he advocated relative to the vesiculæ and their contents: It has been ably done by Meckel in his Manuel of Anatomy, by whom each argument has been seriatim answered, and in most instances, as it appears to me, in a satisfactory manner, but without reference to microscopical research.

Admitting the fact, that the vesiculæ are like the gall-bladder and bladder of urine, recipients, it may be viewed as a fortunate circumstance in our economy, and admirably adapted to the condition of *man*. Like the bile or the urine, so the spermatic fluid in the healthy adult appears to be in constant process of secretion, and to pass as it is formed into its appropriate reservoir, from whence, without disturbance of the system, in a state of continence, it is either pressed out and voided during the act of alvine evacuation, or it may be in part absorbed. Mr. Hunter, in accordance with the opinion which he had formed of the use of the vesiculæ, did not admit this. He believed that the fluid rather accumulated in the testes, and gave rise there to annoyance, requiring its evacuation by a disturbing act,—a doctrine of some danger, especially to youth, in its consequences. In confirmation of what is stated above, and in opposition to the doctrine of Hunter, I may farther state, that I have frequently examined microscopically the fluid from the urethra following the alvine evacuation, and I have always found it, from a healthy person, abounding in animalcules, the majority of which have always been dead; and thus perhaps, seeming to indicate that the vesiculæ are *cloacæ* as well as reservoirs, and are essentially designed for man, to enable him to control and to exercise that moral check on the passions, by which he should be distinguished from brute animals, and without which no considerable advance can be made in civilization, or in elevation of individual condition and character.

Relative to the effects of disease on the fluid of the *vesiculæ seminales*, and on the spermatic fluid generally, the instances brought forward are too few to admit of extensive induction. They seem to show, 1st, That chronic wasting diseases, terminating in death, arrest the secretion of the testes, or the production of those animalcules, on which, there is much reason to infer, the active power of the semen depends; 2dly, That the contents of the vesiculæ and *vasa deferentia*, under the influence of disease, retain longer their characteristic qualities than the contents of the tubuli; and, 3dly, That there is least fluid in the vesiculæ and in the *vasa deferentia*, and that it is most altered in instances of chronic diseases of the abdominal viscera, and especially of the intestines.

The subject of inquiry is in many respects curious, and some other of its relations are not uninteresting or unimportant. I shall allude to one only before concluding, and that connected with medical jurisprudence.

Admitting that spermatic animalcules are characteristic of and essential to healthy spermatic fluid, in certain doubtful criminal cases, probably, decisive evidence may be obtained by means of microscopical examination. The spermatic fluid undergoes change rapidly when exposed to the air, and even soon becomes putrid; but the spermatic animalcules, I find, resist change in a remarkable manner. In one instance, distinct remains of these animalcules were observed in putrid fluid, which had been kept ten weeks, at a temperature varying between 50° and 60° of Fahrenheit. In another instance, some fluid of the vesiculæ was applied to linen, and wrapped in paper and put by in a close drawer. It was examined the following day; at the end of a week, and after eighteen days, and each time animalcules were discovered under the microscope. The mode of making the trial was by saturating a small portion of the smeared linen with a few drops of water, and gently pressing out a drop for the experiment. Fragments of the animalcules were very distinct, and sufficiently characteristic; and on careful inspection, an entire animalcule, here and there, was observed. The application of these facts to the purposes of evidence does not require any comment."

---

LIABILITIES OF THE POPULATION OF ENGLAND AND FRANCE TO HERNIA.*

From an able statistical paper by Mr. Marshall, Deputy Inspector General of Army Hospitals, on Hernia, we extract the following summary of the numbers rejected as conscripts or recruits, on account of Hernia or a tendency to it, in Dublin, Edinburgh, Glasgow, and France.

| | Tables. | No. examin. | Total reject. | Ratio per 1000 reject. | No. rejected by hernia. | Millesimal ratio of rejections in consequence of hernia. |
|---|---|---|---|---|---|---|
| Dub. depot, | I. | 42,740 | 10,279 | 240 | 920 | 21.5 = 1 in 11 of No. reject. |
| Glasgow & Edinburgh, | III. | 9,528 | 2,375 | 248 | 69 | 7.1 1 in 34 do. |
| Germ. Leg. | IV. | 40,462 | 0 | 0 | 365 | 9 |
| France, mean 3 yrs. | V. | 126699 | 46,669 | 368 | 3,948 | 31.2 1 in 11.8 do. |
| Depart. of Seine. | VI. | 26,088 | 11,148 | 427 | 834 | 31.9 1 in 13.3 do. |

" The above summary presents several very remarkable results, one of which is, that the ratio of rejections on account of hernia in Dublin is three times that of Glasgow and Edinburgh. Hernia appears to be 50 per cent. higher among conscripts in France than among recruits examined in Dublin. The uniformity of the ratio of rejections for hernia among conscripts in France for three years (31.2), and in the department of the Seine for a period of eleven years, (31.9), is sufficiently remarkable. The much higher ratio of rejections for disabilities in general among the conscripts in France, than among recruits examined for the periods specified in Dublin and Scotland, is also calculated to excite attention. Many speculative attempts have been made to estimate the proportion of cases of hernia among the populution in this country, but hitherto, as might have been anticipated, without any approximation to a satisfactory conclusion. The records of hospitals, dispensaries, rupture societies, dissecting rooms, and the reminiscences of medical practitioners, do not furnish adequate materials for deducing either the absolute or relative ratio of individuals affected with hernia among the male population of a country; and I may add, that the words 'prevalent,' 'frequent,' or 'not frequent,' when applied to statistical results, and especially in regard to the existence of such an infirmity as rupture, without reference to the number of persons indiscriminately examined, convey no precise or satisfactory information."

---

* Ibid.

